# Oral Health Preventive Knowledge and Behaviors in Relation to Caries Status Among Iraqi Dental Students: A Cross-Sectional Study

**DOI:** 10.7759/cureus.109581

**Published:** 2026-05-25

**Authors:** Hanan Alautry

**Affiliations:** 1 Department of Pediatric and Preventive Dentistry, Wasit University, Kut, IRQ

**Keywords:** cross-sectional study, dental caries, dental students, oral health behaviour, oral health knowledge

## Abstract

Introduction

Knowledge and attitudes toward oral health, shaped by personal experiences, social context, family traditions, life events, and health education programs, are fundamental determinants of oral health behaviors. This relationship is mediated by beliefs, values, and skills, whereby individuals with sufficient knowledge and positive attitudes are more likely to engage in effective self-care practices.

Aim

This study aims to assess the relationship between dental students’ oral health knowledge and behaviors and their caries level.

Materials and methods

The study subjects in this cross-sectional study were 142 fifth-year dental students attending the College of Dentistry, Wasit University, Kut, Iraq. Their data were gathered using a questionnaire that encompassed oral health-related behaviors and validated questionnaires reported in a previous study. Furthermore, caries experience, including decayed, missing, and filled teeth and surfaces (DMFT/DMFS), was recorded for each subject based on the World Health Organization (WHO)-recommended method. The statistical analysis included an independent t-test and bivariate and multivariate Poisson regressions.

Results

The mean age of the study participants was 23 (±1.41) years. Around half of the dental students had low oral health preventive knowledge, brushed their teeth ≥2 times a day, and visited a dentist within the last six months. The analysis showed that there was an association between higher DMFT mean scores and a low level of oral health knowledge (adjusted RR = 1.23; 95% CI: 1.08 to 1.41) and undesirable oral health behaviors, especially among students who did not floss once a day (adjusted RR = 1.30; 95% CI: 1.07 to 1.57).

Conclusion

The findings of this investigation demonstrated the positive effects of oral health knowledge and desirable oral health behaviors on the caries level of dental students.

## Introduction

Knowledge and attitudes toward oral health, shaped by personal experiences, social context, family traditions, life events, and health education programs, are fundamental determinants of oral health behaviors [1,2]. This relationship is mediated by beliefs, values, and skills, whereby individuals with sufficient knowledge and positive attitudes are more likely to engage in effective self-care practices [3].

According to recent estimates by the World Health Organization (WHO), oral diseases affect approximately 3.5 billion people worldwide, including nearly 2 billion individuals with dental caries in permanent dentition and over half a billion children with caries in primary dentition, most of whom reside in middle-income countries [4]. These findings focus on the imperative need to reorganize dental care systems toward preventive approaches, according to WHO recommendations [5]. Risk behaviors, including the frequent consumption of sugary foods and beverages, improper toothbrushing, and irregular dental visits, play an important role in the development of oral diseases. Commonly, these behaviors originate in childhood or adolescence, when a person starts to gain autonomy and assume responsibility for their oral health [6,7]. Consequently, to prevent oral diseases and enhance oral health, local health officials are advised to prioritize preventive oral healthcare programs [8].

Dental professionals are expected to develop and maintain sufficient knowledge and positive attitudes throughout their careers. Measuring these attributes regarding preventive dental care is particularly important because they play a vital role in improving public health through early prevention and reducing the prevalence of dental disease [9]. Several studies report varying levels of knowledge and attitudes toward preventive dentistry among undergraduate dental students [10-12]. From a professional and practical perspective, dental students are presumed to maintain good oral health, since they are future key promoters of oral health within the community [13].

In Iraq, the dentist-to-population ratio is about 2.6 per 10,000 inhabitants, together with an increasing demand for an expanded dental workforce [14]. Nevertheless, dental caries remains highly prevalent, with at least one-third of the population affected by untreated dental decay [15]. Significantly, evidence on the effects of preventive dental health knowledge and behaviors on dental caries status among Iraqi senior dental students remains limited. Accordingly, this study aims to assess the association between dental students’ oral health knowledge and behaviors and their caries level.

## Materials and methods

The current study corresponds with the STROBE standards [16]. Participants for this investigation were 142 fifth-year dental students attending the College of Dentistry, Wasit University, Kut, Iraq, during the 2024-2025 and 2025-2026 academic years. The required sample size for the study was estimated using G*Power software (version 3.1.9.7; Heinrich-Heine-Universität Düsseldorf, Düsseldorf, Germany) [17]. Based on a medium effect size of 0.25, a significance level (α) of 0.05, and a statistical power of 0.80, the minimum required sample size was determined to be 128 participants. A total of 142 Iraqi dental students were enrolled, surpassing the minimum required sample size and ensuring sufficient statistical power to identify significant associations between oral health preventive knowledge, behaviors, and caries status. Subjects were randomly selected from a total of 260 students using a numbering system, and written informed consent was obtained from each student. Participation was optional, and confidentiality was ensured.

The current study was approved by the ethical committee of Wasit College of Dentistry (Reference number: 232025) on February 11, 2025. The questionnaires used in this study included, first, a focus on domains of the students' oral health behaviors. A preferable level of behaviors was defined as toothbrushing at least two times a day, using fluoridated dentifrices, flossing a minimum of once a day, intake of sugary snacks less than once a day, and having a dental appointment within the past six months for a routine check-up.

Second, based on a previously validated questionnaire used in an earlier study [18], 12 items were included to assess dental students’ knowledge of oral health status and preventive dental care, as presented in Appendix 1. Answers were combined to obtain a total knowledge score for each subject, ranging from 12 to 60. Participants were categorized as having high knowledge (scores ≥48), while scores below 48 indicated low knowledge [18].

Moreover, data collection included a clinical oral examination conducted in line with WHO criteria [19]. Caries lesions were evaluated using the decayed, missing, and filled teeth (DMFT) index, plus surfaces (DMFS), for each respondent. Before the main survey, an examiner (HA) calibration exercise was conducted, in which caries status was independently assessed in a group of 20 dental students from the College of Dentistry, Wasit University, on two separate occasions with a two-week interval. These individuals were excluded from the final analysis. Intra-examiner reliability was evaluated using the kappa statistic, which exceeded 0.8 for all clinical parameters, indicating acceptable agreement [20]. Following completion and review of the questionnaires, oral examinations were carried out using WHO periodontal probes (Dentirak) with a 0.5-mm ball tip and disposable mouth mirrors. Examinations were conducted while participants were seated on a dental chair under electric illumination from the dental unit light.

The independent t-test was utilized to compare variables according to mean DMFT scores. Bivariate Poisson regression analyses were applied to investigate the association between predictors and mean DMFT scores. Multivariate Poisson regression analyses were carried out to determine the adjusted effect of students' oral health knowledge and behaviors on mean DMFT scores. Overall, statistical analyses were conducted using a statistical significance threshold of p < 0.05. The data were analyzed using IBM SPSS Statistics for Windows, Version 25 (Released 2017; IBM Corp., Armonk, NY, USA).

## Results

**Figure 1:**
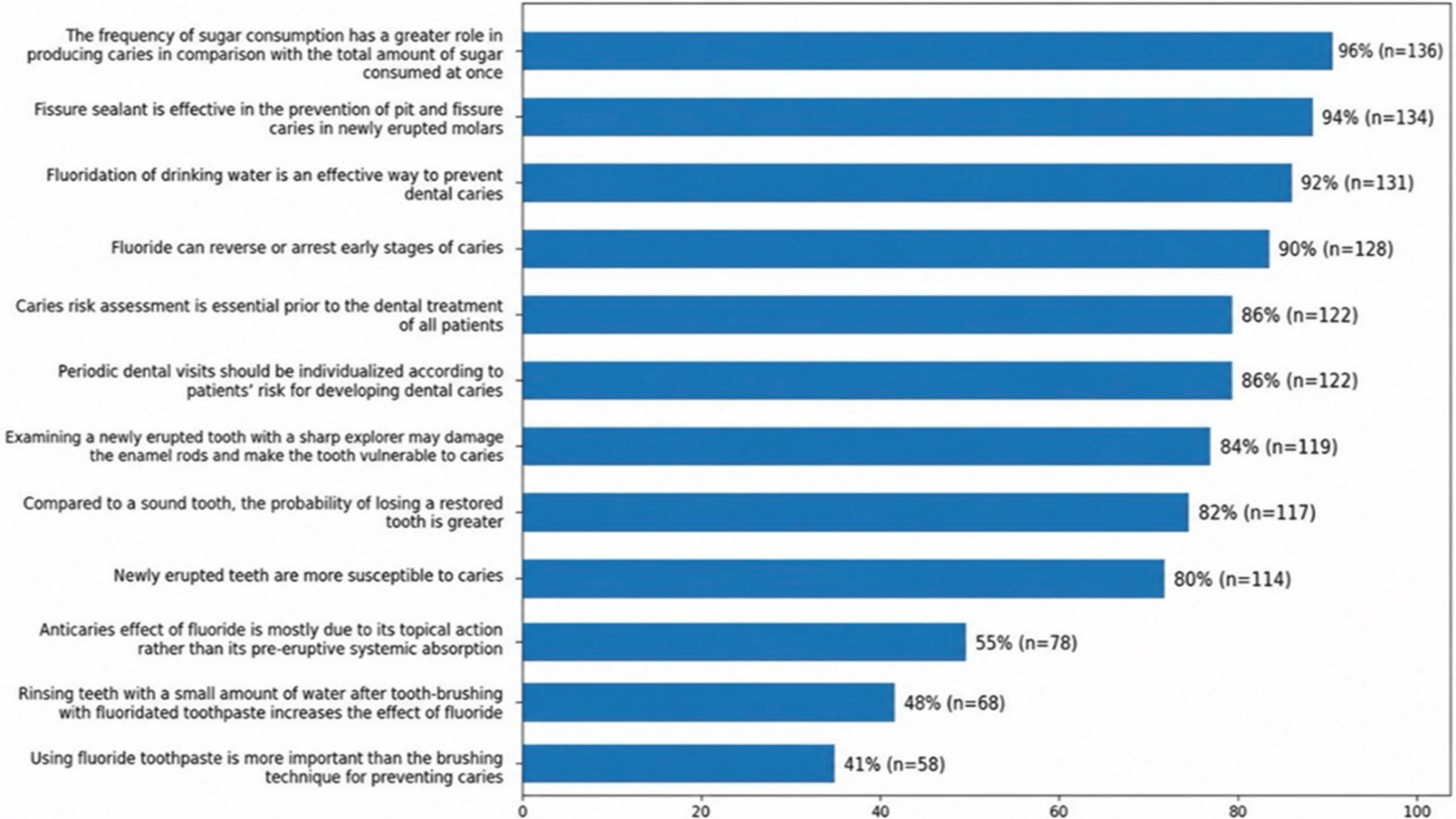
Percentage of dental students who agreed or strongly agreed with statements on dental caries prevention

A total of 142 dental students, 64% of whom were female (n = 91), participated in the study. The proportion of dental students who agreed or strongly agreed with statements about oral health preventive knowledge is shown in Figure 1. About 45% (n = 64) of the subjects were classified as having a high level of knowledge pertaining to the prevention of dental caries. No statistically significant differences in knowledge scores were observed between males and females. Approximately 9 out of 10 dental students recognized the caries-preventive effectiveness of fluoridated drinking water, reducing the intake of sugar-containing foods or drinks, the application of fissure sealants, and the ability of fluoride to arrest or reverse early-stage dental caries. About 40% (n = 57) of participants regarded fluoridated toothpaste as more important for caries prevention than toothbrushing technique.

**Figure 2:**
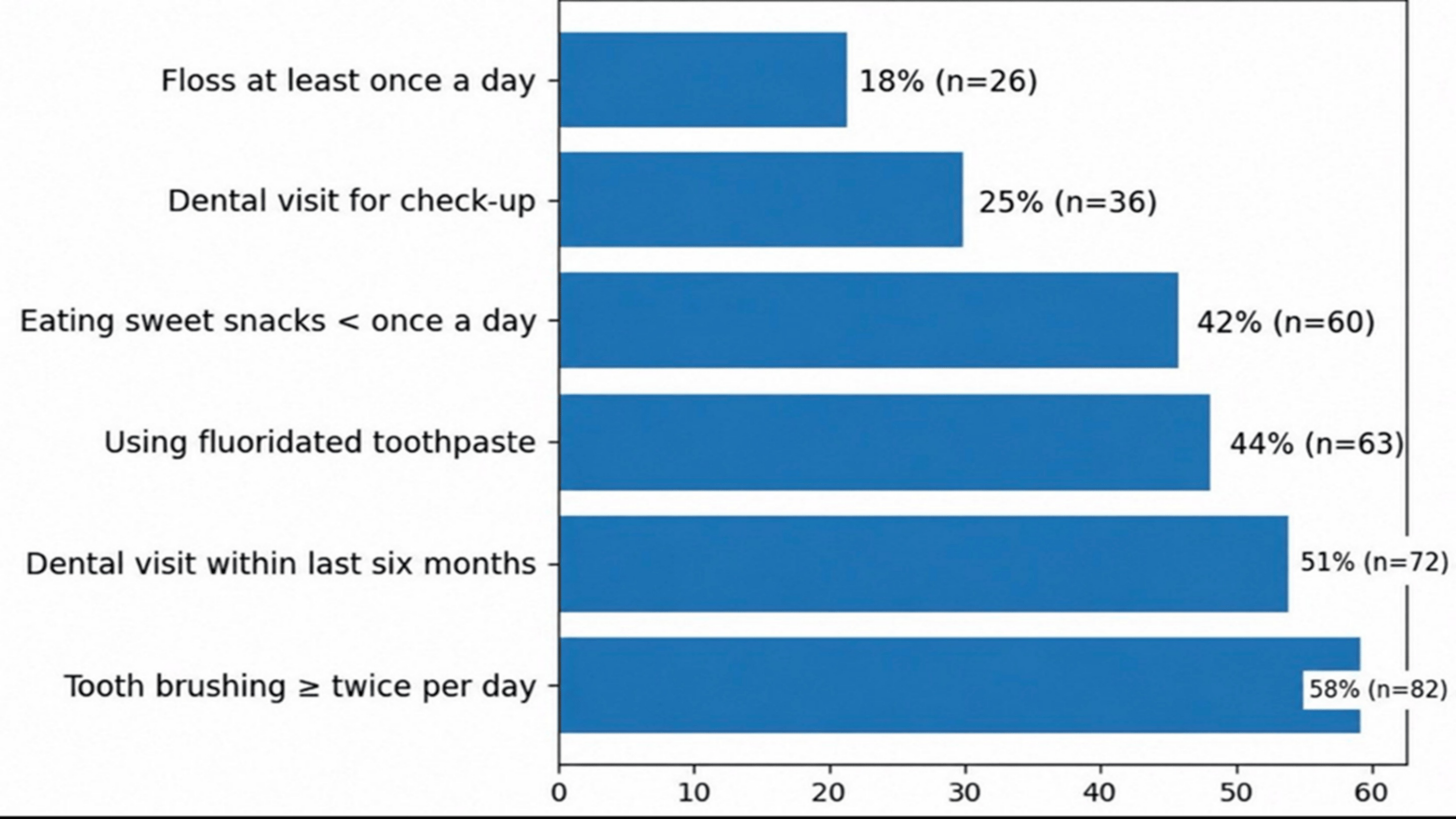
Distribution of Iraqi dental students according to desirable oral health behaviors

Figure 2 shows the distribution of the study participants according to desirable behaviors. Almost half of the participants brushed their teeth ≥ twice a day and visited a dentist within the last six months. Less than 20% (n = 28) of the dental students flossed at least once per day.

**Figure 3:**
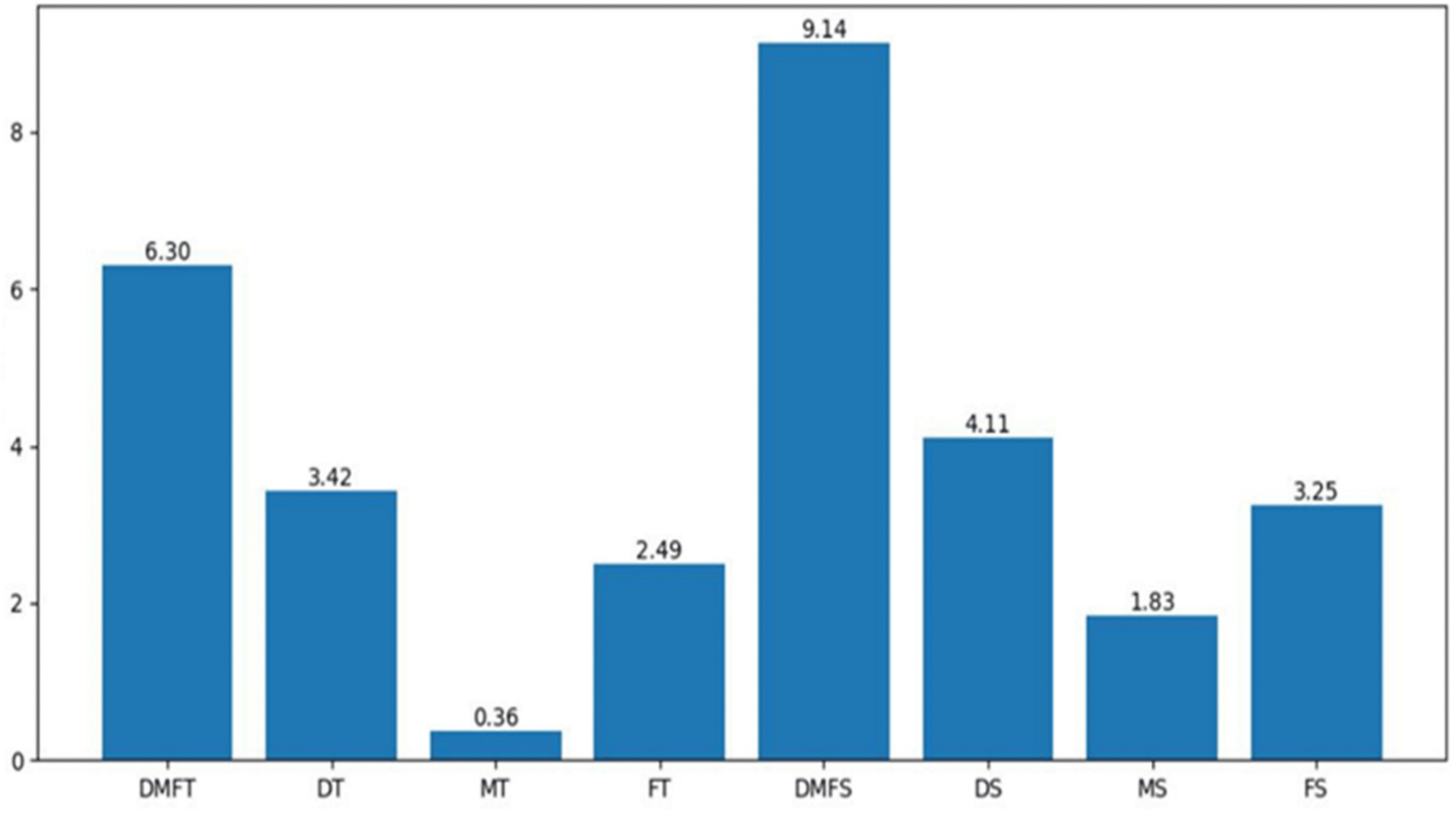
Mean scores of DMFT/DMFS and their components among Iraqi dental students DMFT/DMFS: decayed, missing, and filled teeth/surfaces

Figure 3 displays the caries status within study participants, indicating that the mean scores of the DMFT and DMFS indices were 6.30 (±3.55) and 9.14 (±5.89), respectively. Importantly, the decayed component accounted for a substantial proportion of the indices.

Table 1 demonstrates the association between caries status and oral health knowledge and behaviors. Dental students with high oral health knowledge, who brushed their teeth twice or more per day, flossed at a minimum of once per day, and consumed sugary snacks less than once a day, had significantly lower mean DMFT scores (p < 0.05).

**Table 1:** Relationship of oral health knowledge and favorable oral health behaviors with dental caries status *Statistical evaluation by the independent sample t-test.

Variables		Mean (SD) DMFT	p-value*
Oral health knowledge	High	5.60 (3.06)	0.029*
	Low	6.88 (3.83)	
Tooth brushing ≥ twice per day	Yes	5.52 (2.83)	0.002*
	No	7.38 (4.14)	
Using fluoridated toothpaste	Yes	6.05 (1.92)	0.747
	No	6.34 (3.73)	
Floss at least once a day	Yes	5.01 (2.28)	0.037*
	No	6.60 (3.73)	
Eating sweet snacks less than once a day	Yes	5.35 (3.00)	0.004*
	No	7.01 (3.77)	
Dental visit within the last six months	Yes	6.22 (3.13)	0.789
	No	6.38 (3.94)	
Dental visit for a check-up	Yes	5.52 (4.56)	0.127
	No	6.57 (3.12)	

Table 2 revealed five variables as possible factors associated with the mean DMFT scores, which were included in a multivariate Poisson regression model. Students with low oral health knowledge were associated with higher DMFT mean scores (RR = 1.27; 95% CI: 1.07 to 1.40). For oral health behaviors, Dental students who didn’t brush their teeth twice or more per day (RR = 1.33; 95% CI: 1.17 to 1.52), didn’t floss at least once a day (RR= 1.32; 95% CI: 1.09 to 1.59), didn’t eat sweet snacks less than once a day (RR = 1.31; 95% CI: 1.14 to 1.50), and didn’t visit the dentist for checkup (RR= 1.19; 95% CI: 1.01 to1.31) were associated with higher DMFT mean scores.

**Table 2:** Effects of gender, oral health knowledge, and behaviors on DMFT score of Iraqi dental students using bivariate and multivariate Poisson regression analysis models DMFT: decayed, missing, and filled teeth

Variables	Crude RR	95% CI	p-value	Adjusted RR	95% CI	p-value
Gender						
Male	1	-	-	-	-	-
Female	1.07	(0.88-1.28)	0.471	-	-	-
Oral health knowledge						
High	1	-	-	1	-	-
Low	1.22	(1.07, 1.40)	0.003	1.23	(1.08, 1.41)	0.002
Tooth brushing ≥ twice per day						
Yes	1	-	-	1	-	-
No	1.33	(1.17, 1.52)	<0.001	1.22	(1.05, 1.41)	0.007
Using fluoridated toothpaste						
Yes	1	-	-	-	-	-
No	0.95	(0.78, 1.16)	0.464	-	-	-
Floss at least once a day						
Yes	1	-	-	1	-	-
No	1.32	(1.09, 1.59)	0.003	1.30	(1.07, 1.57)	0.007
Eating sweet snacks less than once a day						
Yes	1	-	-	1	-	-
No	1.31	(1.14, 1.50)	<0.001	1.17	(1.01, 1.37)	0.034
Dental visit within the last six months						
Yes	1	-	-	-	-	-
No	0.97	(0.85, 1.11)	0.704	-	-	-
Dental visit for a check-up						
Yes	1	-	-	1	-	-
No	1.19	(1.01, 1.31)	0.031	1.18	(1.00, 1.38)	0.039

Table 2 explains the results of the multivariate Poisson regression analysis. The analysis showed that there was still an association between the DMFT mean scores and oral health knowledge (adjusted RR = 1.23; 95% CI: 1.08 to 1.41) and oral health behaviors, especially among students who didn’t floss once a day (adjusted RR = 1.30; 95% CI: 1.07 to 1.57).

## Discussion

This investigation assessed the level of oral health preventive knowledge and behaviors of Iraqi dental students and explored their relationship with dental caries status. This study demonstrated a significant relationship whereby dental students with lower preventive care knowledge and undesirable oral health behaviors exhibited approximately 1.2-fold higher caries experience than those with higher knowledge and desirable behaviors. Our study demonstrated that dental students with a lower level of oral health preventive knowledge recorded significantly higher mean scores on the dental caries indices. This is in agreement with previous studies conducted in Pakistan [21] and India [22-24]. Knowledge enhancement attained through oral health education leads to better clinical oral health outcomes, such as improved oral hygiene status and decreased prevalence of dental caries [25].

This study indicated that nearly 90% of students recognized the effectiveness of fluoridated drinking water, reduction of sugary intake, and the application of fissure sealants in preventing dental caries. However, the importance of topical fluoride seems to be undervalued. These findings are consistent with the results of an earlier study carried out in Iraq [18]. Respondents’ attitudes toward preventive dentistry are mainly reflected in their endorsement of its value to society and its role as a crucial component of clinical dentistry. Our results showed that 45% of dental students had high knowledge of preventive dentistry. A similar deficiency in knowledge, particularly regarding topical fluoride, has been reported in studies involving dental students from Iran and Nigeria [26,27]. Prior studies have demonstrated that tailored educational interventions can successfully improve knowledge and promote more positive attitudes among dental students [28,29].

Findings of the current investigation demonstrated that participants with desirable oral health-related behaviors had lower caries levels, highlighting the pivotal role of education and preventive practices in controlling dental caries. These findings are consistent with prior studies conducted in Pakistan [21] and India [30]. In the current investigation, about half of the dental students brushed their teeth twice a day, which is in agreement with a previous study conducted in Serbia [31]. Our findings indicate that the average DMFT among dental students was 6.30 (±3.55), which contrasts with previous reports from India [30] and Pakistan [21]. Overall, variations in clinical oral health outcomes reported across different studies can largely be explained by differences in geographic settings, demographic profiles, and the socioeconomic backgrounds of the studied populations.

This study exhibits distinct advantages. Students' oral health preventive knowledge was assessed using a tool previously validated for use in the local setting [18]. To identify the predictors that impact dental caries status, the analysis relied on the relative risk and the corresponding confidence interval estimates obtained from multivariate Poisson regression models.

This study has certain limitations. The study was conducted in a single academic institution, which may limit the generalizability of the findings. Moreover, the cross-sectional design introduces a snapshot of one point in time, preventing evaluation of causal relationships. Oral health knowledge and behaviors were self-reported, with a possible risk of response bias. 

## Conclusions

The findings of this study showed that dental students with low oral health knowledge and negative oral health-related behaviors had worse dental caries outcomes. A shortage of knowledge related to topical fluoride use in dental caries prevention highlights the need for greater attention to this issue in dental education. Thus, reinforcing knowledge acquisition and encouraging positive attitudes toward preventive dentistry among dental professionals can be achieved by implementing targeted educational strategies within undergraduate and postgraduate academic dental programs.

## Appendices

Appendix 1

This study used part of the questionnaire developed by Alsheekhly et al. (2024) [18], published under a Creative Commons Attribution (CC BY 4.0) Open Access license, allowing reuse with appropriate attribution. Additionally, permission was obtained from the original publisher.

Students' questionnaire about oral health preventive measures

Student name ----------

Student stage --------------

Gender --------------

Is one of your parents a dentist? Yes …………..., No ………….

I) Fluoridation of drinking water is an effective way to prevent dental caries.

1. Strongly agree

2. Agree

3. Disagree

4. Strongly disagree

5. Don't know

II) The frequency of sugar consumption has a greater role in producing caries in comparison with the total amount of sugar consumed at once.

1. Strongly agree

2. Agree

3. Disagree

4. Strongly disagree

5. Don't know

III) Fissure sealant is effective in the prevention of pit and fissure caries in newly erupted molars.

1. Strongly agree

2. Agree

3. Disagree

4. Strongly disagree

5. Don't know

IV) Compared to a sound tooth, the probability of losing a restored tooth is greater.

1. Strongly agree

2. Agree

3. Disagree

4. Strongly disagree

5. Don't know

V) Rinsing teeth with a less amount of water after tooth-brushing with fluoridated toothpaste increases the effect of fluoride.

1. Strongly agree

2. Agree

3. Disagree

4. Strongly disagree

5. Don't know

VI) Examining a newly erupted tooth with a sharp explorer may damage the enamel rods and make the tooth vulnerable to caries.

1. Strongly agree

2. Agree

3. Disagree

4. Strongly disagree

5. Don't know

VII) Using fluoride toothpaste is more important than the brushing technique for preventing caries.

1. Strongly agree

2. Agree

3. Disagree

4. Strongly disagree

5. Don't know

VIII) Caries risk assessment is essential prior to the dental treatment of all patients.

1. Strongly agree

2. Agree

3. Disagree

4. Strongly disagree

5. Don't know

IX) Periodic dental visits should be individualized according to patients’ risk for developing dental caries

1. Strongly agree

2. Agree

3. Disagree

4. Strongly disagree

5. Don't know

X) Fluoride can reverse or arrest the early stages of caries.

1. Strongly agree

2. Agree

3. Disagree

4. Strongly disagree

5. Don't know

XI) Newly erupted teeth are more susceptible to caries.

1. Strongly agree

2. Agree

3. Disagree

4. Strongly disagree

5. Don't know

XII) Anticaries effect of fluoride is mostly due to its topical action rather than its pre-eruptive systemic absorption.

1. Strongly agree

2. Agree

3. Disagree

4. Strongly disagree

5. Don't know

XIII) How often do you usually brush your teeth?

1. Irregularly or never

2. Once a week

3. A few (2-3) times a week

4. Once a day

5. More than once a day

XIV) Do you use a toothpaste containing fluoride when brushing?

1. Always or almost always

2. Quiet often

3. Seldom

4. Not at all

XV) How often do you floss your teeth?

1. Not at all

2. Occasionally

3. A few (2-3) times a week

4. Once in a day

5. More than one time in a day

XVI) How often do you eat sugar-containing snacks or drinks between your main meals?

1. About 3 times a day or more

2. About twice a day

3. About once a day

4. Occasionally; not every day

5. Rarely or never eat between meals

XVII) When was your last dental visit?

1. Within the last 6 months

2. More than 6 months to one year ago

3. More than 1 to 2 years ago

4. More than 2 to 5 years ago

5. More than 5 years ago

6. Never

7. Do not remember

XVIII) What was the reason for your last dental visit?

1. check-up

2. dental pain

3. dental treatment

4. continuation of dental treatment

XIX) Do not remember
